# Development of the AIIMS Bhopal Pre-sternal Keloid Scale (ABPSKS): A Tool for Assessing Severity and Comparing Treatment Outcomes

**DOI:** 10.7759/cureus.76255

**Published:** 2024-12-23

**Authors:** Ved Prakash Rao Cheruvu, Manal M Khan, Anusha V Devalla

**Affiliations:** 1 Burns and Plastic Surgery, All India Institute of Medical Sciences Bhopal, Bhopal, IND; 2 Obstetrics and Gynaecology, All India Institute of Medical Sciences Bibinagar, Hyderabad, IND

**Keywords:** chest keloids, patient satisfaction, pre-sternal keloids, recurrence, scar assessment scales

## Abstract

Pre-sternal keloids are a distinct clinicopathological entity differing in many ways from keloids occurring elsewhere, as there are differences in pathogenesis, clinical presentation, and management. We have reviewed commonly used assessment scales like the Vancouver Scar Scale, the Patient and Observer Scar Assessment Scale, and the Detroit Keloid Scale and identified some shortcomings in applying them in assessing pre-sternal keloids. Therefore, we developed the AIIMS Bhopal pre-sternal keloid scale (ABPSKS), a pre-sternal keloid-specific tool to enable the assessment of the severity of the disease, the efficacy of treatment, and the comparison of outcomes of different treatment modalities. We have incorporated both the patient's and the observer's perspectives in our tool, and we have also incorporated certain measures that are distinct for pre-sternal keloids.

## Introduction

Scar assessment instruments like the Vancouver Scar Scale (VSS), Patient and Observer Scar Assessment Scale (POSAS), and Manchester Scar Scale (MSS) are utilized commonly for assessing keloids. It is worthwhile to note that keloids were not described as a separate entity when these popular scar assessment instruments were designed [1]. The VSS was first reported by Sullivan in 1990. It is the most widely used burn scar assessment scale to evaluate therapies and as an outcome measure in research studies [2]. However, the VSS does not capture the information from the patient's perspective, which is essential as we seek to attain patient satisfaction with outcomes [3].

The POSAS includes the subjective symptoms of the patient and the objective findings taken by an observer. It consists of two components: the patient scale and the observer scale [4]. When POSAS is used to evaluate pre-sternal keloids, we have noted that there are a few shortcomings: (i) the occurrence of recurrent infections, pus discharge, and the formation of multiple sinus tracts in pre-sternal keloids is not given any consideration in POSAS (ii) For a given outcome measure, the method of scoring on a scale of 1 to 10 can become arbitrary and interfere with the reproducibility of measurements (iii) since quality of life is not considered an independent measure, it is difficult to evaluate the impact of the lesion on the overall quality of life of the patient. The MSS was described by Beausang et al. in 1998 [5]. It combines vascularity and pigmentation under the head 'color mismatch' relative to surrounding tissues.

The Detroit Keloid Scale (DKS) does better in addressing some of the above shortcomings [1]. However, it fails to capture some of the features specific to pre-sternal keloids such as the occurrence of multiple sinus tracts and recurrent infections. Keloids are very common in people with skin of color, and it may be difficult to apply the common scar assessment scales, which include measures like vascularity, accurately in all keloids [1]. In an earlier report, we used only certain components of the VSS to evaluate the efficacy of treatment in pre-sternal keloids, as we felt that all the components of VSS were not suited for application in this entity [6].

We believe that pre-sternal keloids should be considered a distinct clinicopathological entity differing from keloids occurring elsewhere as differences exist with regard to pathogenesis, clinical presentation, and management [6]. Therefore, we intended to develop a pre-sternal keloid-specific tool to enable an assessment of the severity of the disease, an assessment of the efficacy of treatment, and to compare outcomes of different treatment modalities.

## Technical report

We reviewed the available scar assessment tools and developed a pre-sternal keloid-specific assessment scale that incorporates all the relevant elements of the patient's and observer's perspectives. Then, we added some additional measures that increased the specificity of the instrument for application in pre-sternal keloids. We named this the AIIMS Bhopal pre-sternal keloid scale (ABPSKS). Similar to other scales, ABPSKS has two components: the patient component and the observer component. The patient component consists of four measures that assess itching, pain, infection/pus discharge, and quality of life on a scale of 0-3. Table 1 shows the patient component of ABPSKS.

**Table 1:** AIIMS Bhopal pre-sternal keloid scale: patient component The total score of the patient component can range from 0-9.

Measure	Score
Itching	
Never	0
Sometimes	1
Frequent, problematic itch	2
Pain	
Never	0
Sometimes	1
Frequent, problematic pain	2
Infection/Pus discharge	
Never	0
Sometimes	1
Frequent, problematic episodes	2
Incessant episodes, no relief	3
Quality of life	
Not affected	0
Somewhat affected	1
Seriously affected	2

The observer component includes measures that assess site, size, thickness, color, pliability, and sinus tracts on a scale ranging from 0-4. Table 2 shows the observer component of ABPSKS.

**Table 2:** AIIMS Bhopal pre-sternal keloid scale: observer component The total score of the observer component can range from 0 -19

Measure	Score
Location	
Remains hidden in seasonally appropriate clothing	0
Remains visible in seasonally appropriate clothing	1
Remains visible in seasonally appropriate clothing and is cosmetically disfiguring	2
Size	
Maximum dimension of the lesion is ≤ 2cm	0
Maximum dimension of the lesion is between 2.1 – 5 cm	1
Maximum dimension of the lesion is between 5.1 – 10 cm	2
Maximum dimension of the lesion is between 10.1 – 20 cm	3
Maximum dimension of the lesion is >20cm	4
Height	
Normal, flat	0
Maximum height of the lesion is < 2 mm	1
Maximum height of the lesion ranges from 2 to < 5 mm	2
Maximum height of the lesion ranges from 5 to < 10 mm	3
Maximum height of the lesion is 10 mm or more	4
Color (over most of the lesion)	
Normal (same as the surrounding skin)	0
Pink	1
Red	2
Purple	3
Brown to black	4
Pliability	
Normal	0
Supple	1
Yielding	2
Firm	3
Sinus tracts	
Absent	0
Present, ≤5 visible sinus openings	1
Present, >5 visible sinus openings	2

The total patient score and the total observer score will be reported separately, and these scores will be used for comparison. Higher scores will represent clinically worse keloids.

## Discussion

We have designed an assessment tool for pre-sternal keloids after reviewing popular scales like VSS, POSAS, and DKS and identifying their shortcomings when applying these in pre-sternal keloids [1,2,4]. The DKS was designed as a keloid-specific outcome instrument for the standardization and comparison of the results of treatments. In ABPSKS, we do not add up the patient component score and the observer component score to derive a total score, as done in DKS [1]. Instead, we express them separately, as we believe that this provides more insight into the patient's and the observer's perspectives than a total score and a severity grading based on the total score. Furthermore, we have incorporated measures scoring for infections/pus discharge and the occurrence of multiple sinus tracts in our tool. To date, these features have not been accounted for in any of the popular scar assessment scales [1,2,4,5]. We have seen these features in our patients with long-standing and large-sized keloids [6]. These features affect the quality of life of the patients. The incorporation of these measures makes our tool more specific for pre-sternal keloids. We intend not to calculate the lesion's surface area as done in DKS, as we believe that it is not only difficult to calculate the surface area of a pre-sternal keloid, but the measurement is also not of much utility [1]. We have observed that the response to treatment in a pre-sternal keloid is more in the dimension of the lesion's height than the lesion's surface area [6]. That is why we have done more sub-classification in the height dimension compared to DKS, which means that small changes can also be captured and the efficacy of treatments can be ascertained accurately [1]. One more significant change is that instead of assessing pigmentation and vascularity of the lesion separately, we have incorporated the measure 'color' of the lesion, similar to MSS [5]. This approach is very useful in patients with skin of color, who form the majority of our cases, where it is difficult to appreciate pigmentation and vascularity separately [6]. We believe that this approach standardizes the assessment of the lesions and makes it easier to apply even to people of color.

## Conclusions

We have designed a pre-sternal keloid-specific assessment tool, the ABPSKS. We believe that this offers an accurate, comprehensive, and disease-specific pre-sternal keloid-specific outcome measure for standardizing and comparing treatment outcomes. Further studies are required to test the validity of this tool and recommend it for broader usage.
